# Reduced gut microbiota diversity in patients with congenital generalized lipodystrophy

**DOI:** 10.1186/s13098-022-00908-8

**Published:** 2022-09-24

**Authors:** Renan Magalhães Montenegro Junior, Clarisse Mourão Melo Ponte, Maria Helane Costa Gurgel Castelo, Alessandro Conrado de Oliveira Silveira, Virgínia Oliveira Fernandes, Catarina Brasil D’Alva, Luiz Felipe Valter Oliveira, Angélica Domingues Hristov, Silviane Praciano Bandeira, Grayce Ellen da Cruz Paiva, José Eduardo Levi

**Affiliations:** 1 University Hospitals, Federal University of Ceará/Ebserh, Fortaleza, CE Brazil; 2 DASA, São Paulo, SP Brazil; 3 Regional University of Blumenau, Blumenau, SC Brazil; 4 BiomeHub, Florianópolis, SC Brazil; 5 Present Address: Christus University Center, CE Fortaleza, Brazil

**Keywords:** Gut microbiota, Diabetes, Lipodystrophy

## Abstract

**Background:**

Previous studies suggest intestinal dysbiosis is associated with metabolic diseases. However, the causal relationship between them is not fully elucidated. Gut microbiota evaluation of patients with congenital generalized lipodystrophy (CGL), a disease characterized by the absence of subcutaneous adipose tissue, insulin resistance, and diabetes since the first years of life, could provide insights into these relationships.

**Methods:**

A cross-sectional study was conducted with patients with CGL (*n* = 17) and healthy individuals (*n* = 17). The gut microbiome study was performed by sequencing the 16S rRNA gene through High-Throughput Sequencing (BiomeHub Biotechnologies, Brazil).

**Results:**

The median age was 20.0 years old, and 64.7% were female. There was no difference between groups in pubertal stage, BMI, ethnicity, origin (rural or urban), delivery, breastfeeding, caloric intake, macronutrient, or fiber consumption. Lipodystrophic patients presented a lower alpha diversity (Richness index: 54.0 versus 67.5; *p* = 0.008). No differences were observed in the diversity parameters when analyzing the presence of diabetes, its complications, or the CGL subtype.

**Conclusion:**

In this study, we demonstrate for the first time a reduced gut microbiota diversity in individuals with CGL. Dysbiosis was present despite dietary treatment and was also observed in young patients. Our findings allow us to speculate that the loss of intestinal microbiota diversity may be due to metabolic abnormalities present since the first years of life in CGL. Longitudinal studies are needed to confirm these findings, clarifying the possible causal link between dysbiosis and insulin resistance in humans.

**Supplementary Information:**

The online version contains supplementary material available at 10.1186/s13098-022-00908-8.

## Background

In recent years, studying the human microbiota and its relationship with health and disease processes has aroused the scientific community’s interest [1–3]. Numerous studies have suggested that intestinal dysbiosis is associated with metabolic diseases, including insulin resistance, diabetes, and obesity [4–6]. Dysbiosis is characterized by loss of microbiota diversity and alteration of its composition, promoting changes in the use and metabolism of diet components by bacteria, with impairment of the mechanisms of protection against invading pathogens [7, 8]. Besides, dysbiosis is associated with endotoxemia and chronic subclinical inflammation, among other mechanisms, which may culminate in predisposition to the development of insulin resistance and obesity [9, 10]. However, the causal relationship between intestinal dysbiosis and metabolic disorders is not fully elucidated despite this evidence.

Although some experimental studies with animal models have demonstrated the existence of biological plausibility for a cause-effect relationship [5, 11], it is not known whether the changes in the intestinal microbiota described in observational studies carried out in humans are due to the metabolic changes themselves or if the changes in the intestinal microbiota precede the appearance of such metabolic diseases. Several factors can influence the human microbiota, including age, genetic predisposition, geographic aspects, birth conditions, dietary habits, physical activity, and drug use [1]. These variables can act as co-founders in the studies available, and these relationships are not yet well established.

Studies on biological models of diseases that are associated with metabolic disorders of monogenic diseases could contribute to a better understanding of the effects of such changes on the intestinal microbiota. In this context, the study of patients with congenital generalized lipodystrophy (CGL), a hereditary disease characterized by the absence of subcutaneous adipose tissue, hypoleptinemia, severe insulin resistance, and diabetes with micro and macrovascular complications [12–15], could provide insights into these relationships. Thus, this study aims to describe the intestinal microbiota of patients with CGL, associating it with the metabolic disorders classically found in this condition.

## Methods

### Study design and patient enrollment

A cross-sectional study was carried out by the Brazilian Group for the Study of Inherited and Acquired Lipodystrophies (BRAZLIPO), Clinical Research Unit, Walter Cantídio University Hospital, Federal University of Ceará (UFC/EBSERH), from October 2019 to March 2020. Patients from the same state in Brazil, Ceará (CE), diagnosed with CGL followed by Federal University of Ceará (*n* = 17), aged between 1 and 40 years old, were included in this investigation. For the diagnosis of lipodystrophy, clinical and molecular criteria were used. The presence of generalized lipodystrophy since birth or early childhood stages was the main criteria for the clinical diagnosis of lipodystrophy. Other characteristics evaluated were acromegalic aspect, apparent muscle hypertrophy, prominent superficial veins (phlebomegaly), hepatomegaly, hypertriglyceridemia, and insulin resistance [16]. For molecular diagnosis, pathogenic variants in *AGPAT2* or *BSCL2* genes were considered for classification in the subtypes of CGL type 1 and CGL type 2, respectively [16]. The description of the main clinical and molecular features of the patients with CGL is provided in Table 1.

**Table 1:** Description of the main clinical features of the patients with CGL (*n* = 17)

Case sex/age/tanner stage	CGL subtype/pathogenic variants	Comorbidities	Drugs	Delivery/breastfeeding (duration)	Dysbiosis
1 Male/1y G1P1	*Type 1 AGPAT2* c.299G&gt;A, p.Ser100Asn (Heterozygosis), c.493-1G&gt;C, p.Leu165_Gln196del (Heterozygosis), and c.589-2A&gt;G, p.Gln196fs*228 (Heterozygosis)	Diabetes	Vitamins Minerals	Abdominal Yes (8 m)	No
2 Male/5y G1P1	*Type 1—AGPAT2* c.299G > A, p.Ser100Asn (Homozygosis); c.493-1G > C, p.Leu165_Gln196del (Homozygosis)	No	No	Abdominal Yes (15d)	Yes
3 Fem/6y M1P2	*Type 1—AGPAT2* c.299G > A, p.Ser100Asn (Homozygosis); c.493-1G > C, p.Leu165_Gln196del (Homozygosis)	Dyslipidemia	No	Vaginal Yes (18 m)	No
4 Male/8y G2P2	*Type 2—BSCL2* c.325dupA, p.Thr109Asnfs*5 (Homozygosis)	Dyslipidemia	Metformin	Vaginal Yes (5 m)	No
5 Male/9y G2P1	*Type 1—AGPAT2* c.299G > A, p.Ser100Asn (Homozygosis); c.493-1G > C, p.Leu165_Gln196del (Homozygosis)	Dyslipidemia	No	Vaginal Yes (6 m)	No
6 Fem/11y M4P4	*Type 2—BSCL2* c.325dupA, p.Thr109Asnfs*5 (Homozygosis)	Diabetes Dyslipidemia Nephropathy Neuropathy Papillary thyroid cancer	Metformin Captopril	Vaginal Yes (5 m)	No
7 Fem/12y M4P5	*Type 1—AGPAT2* c.369_372delGCTC, p.Leu124Serfs*26 (Homozygosis)	No	No	Vaginal Sim (24 m)	Yes
8 Fem/15y M4P5	*Type 2—BSCL2* c.412C > T, p.Arg138* (Homozygosis)	Diabetes Dyslipidemia Nephropathy Neuropathy Liver disease	Metformin Insulin Propranolol	Abdominal Yes (18 m)	No
9 Male/20y G5P5	*Type 2—BSCL2* c.301_302insAA, p.Met101Lysfs*11 (Heterozygosis), c.325dupA, p.Thr109Asnfs*5 (Heterozygosis)	Diabetes Dyslipidemia Neuropathy Nephropathy Retinopathy	Metformin Insulin Metoprolol	Vaginal Yes (36 m)	No
10 Fem/25y M5P5	*Type 1—AGPAT2* c.366–588 + 534del, p.Gly106fs*188	Diabetes Dyslipidemia Nephropathy Neuropathy Hypothyroidism	Metformin Ciprofibrato Enalapril Levothyroxine	Vaginal Yes (24 m)	Yes
11 Fem/25y M5P5	*Type 1—AGPAT2* c.299G > A, p.Ser100Asn (Homozygosis); c.493-1G > C, p.Leu165_Gln196del (Homozygosis)	Diabetes Dyslipidemia Nephropathy	Metformin Insulin Simvastatin	Vaginal No	No
12 Fem/28y M5P5	*Type 1—AGPAT2* c.366–588 + 534del, p.Gly106fs*188	Diabetes Dyslipidemia Nephropathy Retinopathy	Metformin Insulin Rosuvastatin	Abdominal Yes (48 m)	No
13 Fem/31y M5P5	*Type 1—AGPAT2* c.366–588 + 534del, p.Gly106fs*188	Diabetes Dyslipidemia Neuropathy Nephropathy Retinopathy	Metformin Insulina Atorvastatin Omega-3	Abdominal Yes (6 m)	No
14 Fem/31y M5P5	*Type 1—AGPAT2* c.369_372delGCTC, p.Leu124Serfs*26 (Heterozygosis), and c.589-2A > G, p.Gln196fs*228 (Heterozygosis)	Diabetes Hypertension Dyslipidemia Neuropathy Nephropathy Retinopathy Coronary disease	Insulin Rosuvastatin Carvedilol Clopidogrel Gabapentin Fluoxetin Clonazepan Loratadina Hydralazine Ivabradine Tapazol Isosorbide Amlodipine	Abdominal No	No
15 Male/32y G5P5	*Type 1—AGPAT2* c.299G > A, p.Ser100Asn (Homozygosis); c.493-1G > C, p.Leu165_Gln196del (Homozygosis)	Diabetes	Metformin Insulin Aspirin	Vaginal Yes (12 m)	Yes
16 Fem/35y M5P5	*Type 1—AGPAT2* c.646A > T, p.Lys216* (Homozygosis)	Diabetes Hypertension Dyslipidemia Nephropathy	Metformin Insulin Simvastatin Losartan Aspirin	Vaginal Yes (unknown)	No
17 Fem/41y M5P5	*Type 1—AGPAT2* c.299G > A, p.Ser100Asn (Homozygosis); c.493-1G > C, p.Leu165_Gln196del (Homozygosis)	Diabetes Hypertension Dyslipidemia	Metformin Insulin Atorvastatin Losartan	Vaginal Yes (12 m)	No

*CGL* congenital generalized lipodystrophy

For the comparative group, 17 individuals matched for age and sex, non-diabetic, residing in the same state in Brazil, and without pathogenic variants in the *AGPAT2* or *BSCL2* genes were evaluated. Participants who used antibiotics two months before the start of this study with gastrointestinal diseases, liver failure, undergoing bariatric surgery, or using drugs with effects on the immune system were not included in any of the groups. For composition of the comparative group, we asked the volunteers with lipodystrophy and/or their guardians to indicate people from their community. After this indication, the researchers evaluated whether they met the study's inclusion criteria.

### Study protocol

#### Clinical evaluation

Participants were submitted to medical and nutritional interviews and physical examinations. The variables evaluated in our analysis were sex, age, birth conditions, breastfeeding duration, personal and familial history, diagnosis of diabetes, hypertension, dyslipidemia, cardiovascular disease, macrovascular and microvascular complications, use of drugs, dietary habits, anthropometric measures, and blood pressure measurement.

Anthropometric measurements and arterial blood pressure were obtained following the previous recommendation [17]. Tanner’s pubertal classification was used to determine the pubertal stage [18]. Diabetes Mellitus (DM) was diagnosed based on the American Diabetes Association criteria [19]. Dyslipidemia was defined according to the recommendations of the National Cholesterol Education Program Adult Treatment Panel [20] regarding age and sex.

#### Nutritional evaluation

Food intake was assessed using 24-h food recalls collected by a single trained interviewer at two different times with a 15-day interval, according to the Automated Multiple Pass Method (AMPM) [21]. The first interview was in person, and the following interviews were by telephone. The results of the recall analysis were grouped based on the average and were evaluated using the following variables: total caloric value, percentage distribution of macronutrients, including carbohydrates, proteins, saturated fats, polyunsaturated, total cholesterol, and fibers.

#### Laboratory analysis

All blood and urine samples were collected after 10-h fasting. The blood samples were centrifuged at 3000 rpm for 10 min. Subsequently, the serum samples were stored at − 80 °C for further analysis. A biochemical evaluation was performed by determining glycemia, total cholesterol, HDL cholesterol, triglycerides, aspartate aminotransferase, alanine aminotransferase, and urine albumin-creatinine ratio using an enzymatic colorimetric method, according to the manufacturer’s instruction (HITACHI^®^–Roche). Glycated hemoglobin (HbA1c) was dosed by high-performance liquid chromatography (PREMIER^®^–Trinity Biotech). Insulin was determined by electrochemiluminescence (HITACHI^®^–Roche). Leptin was dosed using an enzyme immunoassay (AIKA^®^–Diasorin; REF: CAN-L-4260; analytical sensitivity: 0.5 ng/mL; variation coefficient intra-assay: 3.7–5.0%).

#### Molecular analysis of lipodystrophy

Genomic DNA was extracted from peripheral blood samples using a standard protocol. The entire coding region and the exon–intron boundaries of the *AGPAT2* and *BSCL2* genes were amplified by polymerase chain reaction using intronic oligonucleotide primer pairs (Additional file 1) using a 9700 thermal cycler (Thermofisher). The amplified products were purified using the QIAquick PCR Purification Kit (QIAGEN), followed by a sequencing reaction with the ABI PrismTM BigDye Terminator Kit (Applied Biosystems, Foster City, California, USA). The products of this reaction were subjected to electrophoresis in an ABI Prism 3100 Genetic Analyzer automatic DNA sequencer (Applied Biosystems, Foster City, California, USA). The obtained sequences were aligned with the *AGPAT2* and *BSCL2* reference sequences NG_008090.1 and NG_008461.1, respectively, using the UGENE tool to identify the mutational profile of the participants and their families. The sequence variants found were described according to the variant nomenclature proposed by the Human Genome Variation Society using the transcript reference sequences NM_006412.4 and NM_001122955.3 for the *AGPAT2* and *BSCL2*, respectively.

#### Gut microbiome study

The gut microbiome study was carried out by GeneOne, DASA laboratory (https://geneone.com.br/). Participants were instructed to collect stool samples at their own homes. The samples were collected on two occasions, with an interval of 30 days. The first collection was made during medical care and laboratory tests. Stool samples were placed in a bottle with an appropriate preservative solution [22]. DNA was extracted with QIAGEN DNeasy PowerSoil Kit. Then, amplification of the V3-V4 region from the 16S rRNA gene, with primers 341F (CCTACGGGRSGCAGCAG) [23] and 806R (GGACTACHVGGGTWTCTAAT), was performed [24]. Preparation of the libraries from the PCR product was done with a proprietary protocol (BiomeHub Biotechnologies, Brazil). The libraries were sequenced using the MiSeq Sequencing System (Illumina Inc., USA) and the V2 kit, with 300 cycles and single-end sequencing. The sequences were analyzed using a proprietary pipeline previously described (BiomeHub Biotechnologies, Brazil) [25].

All DNA sequences were evaluated by quality control metrics, using the sum of the probabilities of error of their bases as a base, allowing at most 1% of accumulated error. Subsequently, the DNA sequences corresponding to the Illumina technology adapters were removed. Reads are then analyzed with the Deblur package v.1.1.0 [26] to remove possible erroneous reads, and identical sequences are grouped into oligotypes (clusters with 100% identity). Sequencing clustering with 100% identity provides a higher resolution for the amplicon sequencing variants. Next, VSEARCH 2.13.6 [27] was used to remove chimeric amplicons. We implemented an additional filter to remove amplicon sequence variants (ASVs) below the frequency cutoff of 0.2% in the final sample counts. The remaining ASVs in the samples are used for taxonomic assignment with the BLAST tool [28] against a reference genome database (encoderef16s_rev6, BiomeHub, SC, Brazil). This database is constructed with complete and draft bacterial genomes, focused on relevant bacteria for human microbiota, obtained from NCBI. It is composed of 11,750 sequences, including 1,843 different bacterial taxonomies.

Taxonomies are assigned to each ASVs using the lowest common ancestor (LCA) algorithm. If more than one reference can be assigned to the same ASV with equivalent similarity and coverage metrics (e.g., two distinct reference species mapped to ASV “A” with 100% identity and 100% coverage), the taxonomic assignment algorithm leads the taxonomy to the lowest level of possible unambiguous resolution (genus, family, order, class, phylum, or kingdom), according to similarity thresholds previously established [29].

Alpha diversity of the samples was measured by observed species, Shannon and Simpson index, and relative dominance [30]. The observed species index measures the number of species per sample, defined as “richness.” The relationship between phylum Bacteroidetes and Firmicutes, and the presence of bacteria with pro- and anti-inflammatory profiles were also analyzed.

For the characterization of dysbiosis, a decrease in alpha diversity was considered. However, the presence of bacteria with an anti-inflammatory profile was also considered (*Akkermansia muciniphila, Bifidobacterium *spp.,* Eubacterium rectale, Feacalibacterium prausnitzii, Lactobacillus *spp.,* Prevotella copri, Roseburia *spp., *Veilonella *spp.,* Odoribacter splanchnicus, Coprococcus, Bacteroides cellulosilyticus, Blautia *spp.), as well as the presence of bacteria with pro-inflammatory activity (*Escherichia coli, Klebsiella pneumoniae, Parasutterella *spp.,* Fusobacteriaceae *spp.,* Enterobacter hormaechei, Enterobacter asburiae, Bacteroides caccae, Sutterella wadsworthensis, Bilophila wadsworthia, Ruminococcus gnavus, Fusobacteria *spp.,* Arcobacter butzleri, Bacteroides ovatus, Acinetobacter lwoffii, Clostridium perfringens, Clostridioides difficile, Proteobacteria, Bacteroides vulgatus, Haemophilus parainfluenzae, Enterobacter cloacae, Acinetobacter *spp.).

### Statistical analysis

Data were analyzed using the JAMOVI version 1.6.9 for macOS (Sydney, Australia). Continuous variables were described using the median (25th; 75th), and categorical variables using relative and absolute frequency. The Shapiro–Wilk test evaluated normality. The student’s t-test was used for continuous variables with a normal distribution. Mann–Whitney test was used for continuous variables with a non-parametric distribution. Association between categorical variables was performed using the Chi-square and Fisher’s exact test. Spearman's rank correlation coefficient (r) was calculated for correlation analysis. The level of statistical significance adopted for all tests was 5% (p < 0.05).

## Results

The median age of patients was 20 years old (9.0; 31.0), 64.7% *(n* = 11) were female and 35.3% (*n* = 6) male. There was no difference in age, pubertal stage, ethnicity, or origin (rural or urban) between patients with CGL and healthy individuals. We did not observe differences between the groups regarding birth conditions (parturition type and time, hospitalization, or use of antibiotics in the first 30 days of life) and previous breastfeeding history. Regarding nutritional assessment, the groups did not differ in body mass index (BMI), caloric intake, and macronutrient and fiber intake adequacy (Table 2). Among patients with lipodystrophy, 12 (70%) were diagnosed with diabetes, and 11 (64.7%) were treated with metformin.

**Table 2:** Socio-demographic characterization of the patients

	CGL (*n* = 17)	Healthy (*n* = 17)	*p* value
Female; n (%)	11 (64.7)	11 (64.7)	> 0.999
Age; years	20.0 (9.0; 31.0)	19.0 (10.0; 30)	0.823
Pre-puberty; n (%)	2 (11.8)	5 (29.4)	0.361
Adults (> 18 years-old); n (%)	9 (52.9)	9 (52.9)	> 0.999
Urban area; n (%)	9 (52.9)	11 (64.7)	> 0.999
Pardos; n (%)	9 (52.9)	9 (52.9)	> 0.999
Vaginal discharge; n (%)	11 (64.7)	11 (64.7)	> 0.999
Hospitalar discharge; n (%)	14 (82.3)	16 (94.1)	0.601
Term discharge; n (%)	16 (94.1)	17 (100)	> 0.999
Hospital admission first 30 days of life; n (%)	3 (17.6)	0 (0)	0.227
Antibiotic use in the 30 days of life; n (%)	2 (11.8)	0 (0)	0.325
Exclusive breastfeeding time (months)	3.0 (0.5; 5.5)	5.0 (3; 6.5)	0.080
Total breastfeeding time (months)	10 (5.0; 19.5)	24 (6.0; 24.0)	0.269
BMI (kg/m^2^)	20.7 (18.6; 23.3)	19.0 (17.8; 27.6)	0.601
Total caloric intake (kcal)	1607 (1263; 1819)	1689 (1310; 2320)	0.357
CHO adequacy (%)	10 (58.8)	9 (52.9)	0.781
CHO intake (%)	53.7 (49.3; 56.5)	51.6 (49.0; 55.4)	0.683
Protein adequacy (%)	13 (76.5)	8 (47.1)	0.157
Protein intake (%)	23.8 (20.7; 27.2)	19.6 (17.2; 23.4)	0.048
Total fat adequacy (%)	7 (41.2)	8 (47.1)	0.092
Total fat intake (%)	20.9 (19.5; 28.2)	29.4 (25.5; 31.8)	0.024
Cholesterol (mg)	224 (193; 303)	285 (207; 448)	0.357
Fibers (g)	20.3 (15.5; 29.3)	16.9 (11.4; 28.2)	0.179

Continuous variables were described using the median (25th; 75th), and categorical variables using relative and absolute frequency

*CGL* congenital generalized lipodystrophy, *CHO* carbohydrate

The patients with lipodystrophy presented less diversity, measured by the richness index (54.0 versus 67.5; *p* = 0.008) (Table 3). Among them, four patients (23.5%) had characteristics compatible with intestinal dysbiosis versus only one subject (5.9%) in the group of healthy individuals (*p* = 0.335).

**Table 3:** Microbiome analysis in CGL and healthy individuals

	**CGL (*n* = 17)**	**Healthy (*n* = 17)**	***p* value**
Dysbiosis; n (%)	4 (23.5)	1 (5.9)	0.335
Dominance	0.068 (0.049; 0.085)	0.055 (0.039; 0.093)	0.518
Richness	54.0 (48.0; 59.5)	67.5 (58.0; 79.5)	0.008
Shannon	3.19 (2.79; 3.30)	3.40 (3.02; 3.65)	0.114
Simpson	0.930 (0.914; 0.951)	0.944 (0.906; 0.965)	0.448
Bacteroides to Firmicutes ratio	2.07 (1.02; 2.59)	1.07 (0.74; 3.82)	0.535
Bacteroides plus Firmicutes phyla	0.89 (0.83; 0.94)	0.85 (0.71; 0.92)	0.117
Pro-inflammatory	12 (70.6)	14 (82.3)	0.688
*Akkermansia muciniphila*	3 (17.6)	4 (23.5)	> 0.999

The diversity parameters presented refer to the average of the values obtained in the collection times T0 and T1. Continuous variables were described using the median (25th; 75th), and categorical variables using relative and absolute frequency

*CGL* congenital generalized lipodystrophy

A subanalysis of adult patient data showed that the richness index (54 vs. 70; p = 0.024) was lower in patients with CGL compared to healthy individuals.

There was no difference in the anti- and pro-inflammatory bacteria profiles between groups. We did not observe differences between samples collected at baseline and after 30 days in CGL and healthy groups (Additional file 2). The abundance composition of each bacterium data is presented as Additional file 3.

**Fig. 1:**
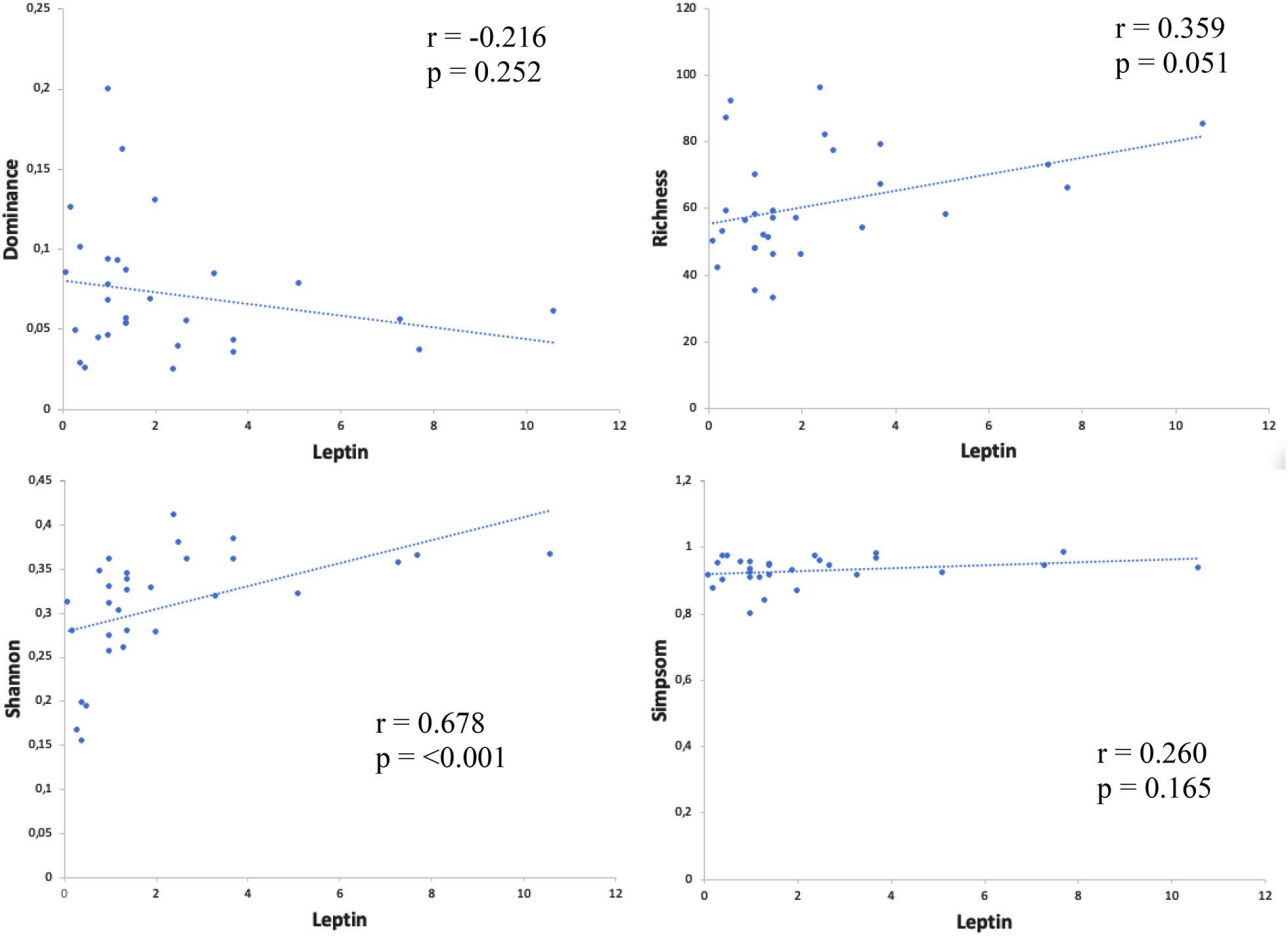
Correlation matrix between leptin levels and gut microbiota diversity parameters in patients with congenital generalized lipodystrophy and healthy individuals (n = 34). There was a positive correlation between leptin levels and Shannon index (r = 0.678; p < 0.001). Spearman's rank correlation coefficient (r) was calculated for correlation analysis. Leptin was dosed using an enzyme immunoassay (AIKA^®^–Diasorin; REF: CAN-L-4260) and expressed in ng/mL

Patients with CGL had higher values of glycated hemoglobin, insulin, triglycerides, and urine albumin-creatinine ratio and lower values of leptin and HDL-c (Additional file 4). There was a positive correlation between leptin levels and Shannon index (r = 0,678; p < 0.001) (Fig. 1). No differences in diversity parameters were observed when analyzing the gender, age, CGL subtype, the presence of diabetes, and the use of metformin or insulin (Additional file 5).

## Discussion

In this study, we evaluated the gut microbiota of patients with CGL and demonstrated a reduction in bacterial diversity in individuals with severe hypoleptinemia and insulin resistance since childhood. Our data is the first to assess the presence of dysbiosis in CGL patients.

Metabolic alterations in CGL are genetically determined and result from mutations in specific genes related to adipogenesis [14]. Our patients present the two most common CGL subtypes, type 1 and type 2 CGL. Type 1 CGL is associated with *AGPAT2* mutations. *AGPAT2* gene is involved in the biosynthesis of glycerophospholipids and triglycerides. Type 2 CGL is caused by *BSCL2* mutations and is considered a more severe form. *BSCL2* gene is involved in the maturation of preadipocytes and adipocytes [12, 13].

The development of insulin resistance and diabetes in these young patients is independent of traditional factors related to the development of metabolic diseases, including diet, physical activity, and obesity. Patients with CGL have an absence of subcutaneous adipose tissue, which is associated with insulin resistance and diabetes mellitus in childhood or adolescence due to severe hypoleptinemia [12–15].

Leptin is a hormone predominantly produced in the adipose tissue and plays a central role in regulating energy metabolism and food intake [31]. In addition to its synthesis by adipocytes, leptin can be produced in smaller amounts by enteroendocrine cells (EEC) of the gastrointestinal (GI) tract [32, 33]. Leptin synthesis by adipose tissue can be regulated by short-chain fatty acids (SCFA), produced through the metabolization of complex carbohydrates by intestinal bacteria. Furthermore, experimental studies have shown that SCFA benefits insulin signaling, improving its peripheral tissue sensitivity [34]. Besides, gastrointestinal microbiota also influences leptin production by EECs [35–37].

It has been demonstrated that patients with CGL present hyperphagia due to reduced leptin [16]. Intestinal microbiota also influences the control of hunger/satiety by producing neurotransmitters such as serotonin, dopamine, and γ-aminobutyric acid (GABA) that can act locally in the enteric nervous system or transmit signals to the central nervous system through vagal afferent neurons. In addition, SCFA, mainly butyrate, acetate, and propionate, can bind to G protein-coupled receptors (GPCR), specifically to GPR41 and GPR43, in EECs, stimulating the release of anorexigenic hormones, such as glucagon-like peptide (GLP-1) and peptide YY (PYY) [34, 38, 39]. The imbalance of the intestinal microbiota can predispose to low-grade chronic inflammation, causing vagal remodeling and changes in the control of hunger/satiety, increasing the food intake [40, 41]. It is interesting to note, however, that most patients with CGL in this study had a normal BMI, consumed a diet with adequate macronutrients and fiber, and still presented microbiota alterations like those observed in previous studies with obese patients [42–44]. Thus, our findings raise the question that the lower diversity of the intestinal microbiota observed in patients with CGL may result from the metabolic alterations themselves.

Leptin can modulate many essential functions in the GI tract, including motility, absorption, growth, and immunity [45]. Leptin receptors are abundant in the GI tract and are located in the afferent and efferent vagus nerve endings [46]. Leptin regulates gastric motility, delaying gastric emptying, and presents a complex effect on the motility of the small bowel [47, 48]. Besides, leptin modulates the absorption of macronutrients in the GI tract [49], stimulates gut mucosal cell proliferation, and inhibits apoptosis [45].

In addition, mice leptin-deficient *ob/ob* or leptin receptor (LepRb)-null *db/db* mice present hyperphagia, obesity, and alterations in the gut microbiota [50, 51]. However, it remains unclear whether compositional changes in the gut microbiota are due to hyperphagia or physiologic changes associated with obesity or from other leptin actions independent of food intake and adiposity. Furthermore, Duggal et al. identified a mutation in the leptin receptor is associated with *Entamoeba histolytica* infection in children, suggesting a role for leptin signaling in the gut epithelium in the host's defense against intestinal pathogens [52].

Gut antimicrobial peptides (AMPs) secreted by Paneth cells represent the central mechanism by which the host influences the gut microbiome [53]. AMPs not only defend against enteric pathogens but also have the capacity to alter the composition of commensal microbes [54]. Rajala et al. suggested that leptin action might modulate bacterial populations within the gut by controlling the expression of AMPs. Their data demonstrated a decreased mRNA expression of gut AMPs in leptin receptor (LepR)-deficient *db/db* mice, suggesting a potential role for LepRb signaling for AMP modulation, independent of food intake, in the host regulation of gut microbiota composition [55].

Our study demonstrated a positive correlation between leptin levels and the Shannon index, a well-known diversity index used in microecological studies. This alpha diversity index is a quantitative indicator of the number of bacteria present in a stool sample, whose value increases when the number of species and the evenness increases—the higher the Shannon index value, the higher the community diversity [56]. Our data, together with the evidence presented in the previous studies by Duggal et al. [52] and Rajala et al. [55], make us speculate that hypoleptinemia could promote changes in the intestinal microbiome in patients with CGL.

Moreover, diabetes and hyperglycemia are also associated with modifications in the gut microbiota [57]. Diabetes can cause gastrointestinal disturbances, mainly associated with microangiopathic complications, including neuropathy. Autonomic neuropathy is related to changes in intestinal motility, leading to reduced intestinal transit, bacterial overgrowth, and microbiota imbalance. Diabetic angiopathy secondary to chronic hyperglycemia may also be associated with intestinal ischemia and the development of diabetic gastroenteropathy [58]. Besides, in animal models, hyperglycemia, through its action on type-2 glucose transporters present in intestinal epithelial cells, can change the integrity of the intestinal barrier by modifying the composition of mucus and the function of tight junctions. This effect promotes increased mucosal permeability, leading to the "leaky gut" associated with bacterial translocation [59]. Although this phenomenon has been associated with dysbiosis, it also may be due to hyperglycemia per se. Thus, in diabetic patients, the profile of bacteria in the intestine may act synergistically with hyperglycemia in developing endotoxemia and systemic inflammation, worsening metabolic disorders.

Many CGL patients in our study were using metformin. It is described that metformin affects intestinal microbiota composition and increases some bacterial species, such as *Lactobacillus spp* and *Akkermnsia mucipniphila* [60–63]. *Akkemansia muciniphila* is a bacterium in the intestinal mucus with a critical barrier function and one of the most relevant producers of SCFAs [64]. Although the use of metformin has been associated with changes in the intestinal bacterial microbiota, we did not observe these findings in our patients, and a reduction of microbiota diversity in CGL patients was observed despite metformin use.

It is also important to consider that the lipodystrophy subtype could influence the microbiome. Patients with CGL have reduced adiponectin levels, especially in type 1 CGL [16]. Adiponectin is an adipokine with an anti-inflammatory function associated with metabolic disturbance [65]. In an experimental study with suckling rats, Grases-Pintó et al. recently demonstrated that adiponectin supplementation might influence microbiota composition [66]. In our study, we found intestinal dysbiosis just in patients with type 1 CGL. However, we did not demonstrate differences in diversity parameters between groups with type 1 and type 2 CGL. As we did not evaluate the adiponectin levels, we could not establish any association between this adipokine and the microbiota diversity parameters in our analysis. The lack of adiponectin measurement is a limitation of our study and should be further explored in future studies.

Also, it is interesting to discuss that impairment of the seipin protein in patients with type 2 CGL could lead to neuronal dysfunction, especially motor neuron disease [67]. Although these diseases can lead to different neurological manifestations, we did not find studies showing changes in intestinal motility related to seipinopathies. Besides, our patients had no clinical signs of motor neuron disease or other neurological signs of seipinopathies.

We did not find any difference in the proportion between Bacteroidetes and Firmicutes phyla in patients with CGL and healthy controls. Although previous experimental studies have shown an increase in the proportion of Firmicutes, which could predispose to the development of obesity and metabolic disease [68, 69], recent studies have questioned these findings [70–72]. The relative abundance of the Bacteroidetes and Firmicutes phyla is highly variable between subjects from the same population. Many factors could influence the composition of the gastrointestinal microbiota, making it difficult to associate the ratio between Bacteroidetes and Firmicutes phyla with determining health status. Currently, although the gut microbiota could contribute to the development of obesity, the evidence suggesting an association between obesity and alterations of the Firmicutes/Bacteroidetes ratio is still questionable [73].

Answering all these questions is not straightforward. Most studies on the assessment of the gut microbiota in humans are subject to numerous biases that act as confounding factors. Here, we tried to control for possible confounders of relevance, such as age, gender, geographic location, ethnicity, birth conditions, breastfeeding, physical activity, and diet. We believe the rigorous selection of the comparative group was a strong point of this research. The main limitation of our study is the sample size; however, considering the rarity of CGL and the number of patients evaluated, our data are relevant to the literature.

CGL is a rare disease, which makes it exceedingly difficult to have a large enough sample size within a particular age group. However, this is critical because the development of the microbiome changes rapidly early in life. Thus, the data were analyzed according to the age group to understand the connections between microbiome and metabolism better. We observed a reduction of alpha diversity even in the sub-analyses of adult patients, a subgroup with a more stable microbiome [1].

We use the term "dysbiosis" to characterize patients with reduced alpha diversity and altered profile of the pro- and anti-inflammatory bacteria [1]. However, we understand that there is too much variability in the definition of dysbiosis. We also emphasize that the main parameters to assess the healthy gut microbiota in this study were the alpha diversity indexes, which give less subjectivity to the analysis, reinforcing the results found.

Our data allow us to hypothesize some inferences about causality between gut microbiota and metabolic disease once we evaluate patients considered as biological models to study the absence of adipose tissue and leptin deficiency. It seems to us that there is a dual pathway in the modulation between the microbiota and metabolic disease. In our patients, a role of hypoleptinemia in the loss of gut microbiota diversity is possible once we observed dysbiosis in patients who still had no hyperglycemia or diabetes. Due to the small sample size and rarity of CGL, we believe the hypotheses generated here could be tested further in animal models or via comparisons to people with type 2 diabetes.

Still, it is interesting to make a parallel with obese patients in this context. Could the hyperleptinemia observed in obese patients modify the microbiota? Is there resistance to leptin in the receptors of the vagus nerve endings in the GI tract? Would this imply changes in intestinal motility and microbiota in obese patients? All these questions are relevant to understanding the relationship between obesity and microbiota. Studying the impact of leptin replacement in the treatment of CGL could establish better inferences about this relationship.

Lastly, it is necessary to consider the potential therapeutic effect of microbiota manipulation on metabolic disease management. Several drugs with prebiotic and probiotic action have been studied with variable effects on metabolic outcomes [74–77]. Besides, advances in engineered bacteria using synthetic biological methods reflect a new possibility for microecological therapy. Experimental studies with animal models of diabetes and obesity have been conducted with incipient but promising results [57]. Understanding the role of microbiota and its metabolites, such as SCFAs, in the leptin synthesis by EECs could result in developing strategies to minimize the repercussions of hypoleptinemia in patients with CGL.

## Conclusion

In summary, this is the first study to demonstrate a reduction of gut microbiota diversity in individuals with CGL. Reduced gut microbiota diversity was present despite dietary treatment and was also observed in young patients. Our findings allow us to speculate that the loss of intestinal microbiota diversity may be due to metabolic abnormalities present since the first years of life in CGL. Longitudinal studies are needed to confirm these findings, clarifying the possible causal link between dysbiosis and insulin resistance in humans.

## Supplementary Information

**Additional file 1. **Oligonucleotide primer pairs used for the amplification of the *AGPAT2* and *BSCL2* coding regions.

**Additional file 2. **Microbiome analysis in each collection time (t0 and t1) by group.

**Additional file 3. **Abundance composition of each bacterium data.

**Additional file 4. **Biochemical and hormonal analysis.

**Additional file 5. **Diversity parameters analyzed by the age, gender, the presence of diabetes, CGL subtype, and the use of metformin or insulin.

## Data Availability

The datasets generated and/or analysed during the current study are not publicly available due individual privacy of the participants could be compromised but are available from the corresponding author on reasonable request.

## Abbreviations

AMP: Antimicrobial peptides
AMPM: Automated multiple pass method
ASV: Amplicon sequence variants
BMI: Body mass index
BRAZLIPO: Brazilian Group for the Study of Inherited and Acquired Lipodystrophies
CGL: Congenital generalized lipodystrophy
DM: Diabetes mellitus
EEC: Enteroendocrine cells
GABA: γ-Aminobutyric acid
GI: Gastrointestinal
GLP-1: Glucagon-like peptide 1
GPCR: G protein-coupled receptors
HbA1c: Glycated hemoglobin
LCA: Lowest common ancestor
PYY: Peptide YY
SCFA: Short-chain fatty acids
