# Supplementary material for: Reduced gut microbiota diversity in patients with congenital generalized lipodystrophy
Source: Diabetol Metab Syndr. 2022 Sep 24;14:136. doi: 10.1186/s13098-022-00908-8 (PMC9508722; doi:10.1186/s13098-022-00908-8)
Supplement: Supplementary file 1 — Additional file 1. Oligonucleotide primer pairs used for the amplification of the AGPAT2 and BSCL2 coding regions. [file 13098_2022_908_MOESM1_ESM.docx]

**Additional file 1**

**Additional file 1.** Oligonucleotide primer pairs used for the amplification of the *AGPAT2* and *BSCL2* coding regions

| ***AGPAT2* Exon** | **Primer sequences** | **Product** |
| --- | --- | --- |
| 1 | 5’-cgcaataaggggcctgag-3’  5’-ggaccccctcctgtgc-3’ | 430 pb |
| 2 | 5’-gggactctgtccgcttca-3’  5’-cagccctgtgtcctcgtc-3’ | 398 pb |
| 3 | 5’-ggtgctcagcagctgtcttc-3’  5’-tttctgccaaaaccaagtcac-3’ | 338 pb |
| 4 | 5’-aaaacaagacccccacatcat-3’  5’-gaggagtcccttgtgtgtcaag-3’ | 406 pb |
| 5 | 5’-cctcagctgtgcgtctcc-3’  5’-gagtcactcattcgccacat-3’ | 240 pb |
| 6 | 5’-ctagggagtccaggggaaga-3’  5’-agtgacagaaggggcttcct-3’ | 472 pb |

| ***BSCL2* EXON** | **Primer sequences** | **Product** |
| --- | --- | --- |
| 1 | 5’-cctcccacggctacaaaag-3’  5’-gagtttctcctctccgcctc-3’ | 294 pb |
| 2 | 5´-tctcgttcctcaaagccagt-3´  5´-tgaggggattgaactgaatga-3´ | 488 pb |
| 3 | 5´-gccctagggggcaaagaa-3´  5´-ccttctctctaggcctttctca-3´ | 243 pb |
| 4 | 5´-aggctacccaagagggttgt-3´  5´-ctcccaaactgctgggatta-3´ | 378 pb |
| 5 e 6 | 5´-ctactcaggggtggttgagg-3´  5´-aacccattacctctgcttgg-3´ | 594 pb |
| 7 e 8 | 5´-cagagccagctgtaaccaaa-3´  5´-cggtgataccctaagcctca-3´ | 566 pb |
| 9 e 10 | 5´-accgactgagacaagggtca-3´  5´-ATCTTCCCAGGAGCCTGAAC-3´ | 475 pb |
| 11 | 5´-TAGAGCCTGAGGCCAGTGAT-3´  5´-cctctaatggcggaaagtga-3´ | 438 pb |
