# Supplementary material for: Reduced gut microbiota diversity in patients with congenital generalized lipodystrophy
Source: Diabetol Metab Syndr. 2022 Sep 24;14:136. doi: 10.1186/s13098-022-00908-8 (PMC9508722; doi:10.1186/s13098-022-00908-8)
Supplement: Supplementary file 2 — Additional file 2. Microbiome analysis in each collection time (t0 and t1) by group. [file 13098_2022_908_MOESM2_ESM.docx]

**Additional file 2.** Microbiome analysis in each collection time (t0 and t1) by group

|  | **CGL** | | | **Healthy** | | |
| --- | --- | --- | --- | --- | --- | --- |
|  | **T0**  **(*n*=17)** | **T1**  **(*n*=17)** | ***p* value** | **T0**  **(*n*=17)** | **T1**  **(*n*=17)** | ***p* value** |
| Dominance | 0.058  (0.044; 0.086) | 0.058  (0.045; 0.095) | 0.587 | 0.052  (0.035; 0.102) | 0.055  (0.043; 0.082) | 0.927 |
| Richness | 56.0  (46.0; 65.0) | 53.0  (47.0; 57.0) | 0.061 | 74.0  (55.0; 79.0) | 70.0  (57.0; 80.0) | 0.776 |
| Shannon | 3.34  (3.08; 3.54) | 3.30  (2.81; 3.41) | 0.263 | 3.64  (3.09; 3.80) | 3.43  (3.26; 3.78) | 0.579 |
| Simpson | 0.942  (0.913; 0.955) | 0.941  (0.905; 0.955) | 0.579 | 0.948  (0.898; 0.964) | 0.945  (0.917; 0.970) | 0.263 |
| Bacteroides to Firmicutes ratio | 1.78  (0.57; 2.83) | 1.47  (1.06; 1.94) | 0.603 | 1.02  (0.56; 1.31) | 1.01  (0.52; 1.72) | 0.747 |

CGL: congenital generalized lipodystrophy

Continuous variables were described using the median (25th; 75th), and categorical variables using relative and absolute frequency.
