# Supplementary material for: Reduced gut microbiota diversity in patients with congenital generalized lipodystrophy
Source: Diabetol Metab Syndr. 2022 Sep 24;14:136. doi: 10.1186/s13098-022-00908-8 (PMC9508722; doi:10.1186/s13098-022-00908-8)
Supplement: Supplementary file 3 — Additional file 3. Abundance composition of each bacterium data. [file 13098_2022_908_MOESM3_ESM.docx]

**Additional file 3.** Abundance composition of each bacterium data

Taxonomy report*:

<https://drive.google.com/file/d/1Q0iVoJRVYyrdv2ji7QlkZdeHqopPp5jT/view?usp=sharing>

Tables:

<https://drive.google.com/drive/folders/13TKc74kxCmfchGZikviVyuiWnJSJ3Obe?usp=sharing>

* Download is required to access the data.
