# Supplementary material for: Reduced gut microbiota diversity in patients with congenital generalized lipodystrophy
Source: Diabetol Metab Syndr. 2022 Sep 24;14:136. doi: 10.1186/s13098-022-00908-8 (PMC9508722; doi:10.1186/s13098-022-00908-8)
Supplement: Supplementary file 4 — Additional file 4. Biochemical and hormonal analysis. [file 13098_2022_908_MOESM4_ESM.docx]

**Additional file 4.** Biochemical and hormonal analysis

|  | **CGL (*n*=17)** | **Healthy (*n*=17)** | ***p* value** |
| --- | --- | --- | --- |
| Plasmatic glucose (mg/dL) | 89 (71; 190) | 76 (73.0; 83.0) | 0.117 |
| Glycated hemoglobin (%) | 6.7 (5.1; 9.9) | 5.4 (5.1; 5.5) | 0.040 |
| Basal insulin (mUI/mL) | 32.2 (15.6; 41.4) | 9.3 (7.1; 14.1) | <0.001 |
| Total cholesterol (mg/dL) | 158 (143; 172) | 145 (134; 166) | 0.293 |
| HDL-c (mg/dL) | 33.0 (24.5; 37.0) | 53.0 (45.0; 65.0) | <0.001 |
| LDL-c (mg/dL) | 88.5 (75.0; 102) | 68.6 (52.6; 89.8) | 0.200 |
| Triglycerides (mg/dL) | 258 (121; 426) | 66.0 (61.0; 93.0) | <0.001 |
| AST (U/L) | 23 (20.0; 39.0) | 30.0 (24.0; 36.0) | 0.501 |
| ALT (U/L) | 39.0 (21.0; 48.0) | 17.0 (13.0; 25.0) | 0.002 |
| ACR (mg/g) | 406 (84.1; 2609.0) | 9 (4.3; 13.1) | <0.001 |
| Leptin (ng/mL) | 1.1 (0.8; 1.7) | 2.5 (1.3; 4.4) | 0.015 |

CGL: congenital generalized lipodystrophy; HDL-c: high density lipoprotein; LDL-c: low density lipoprotein; AST: aspartate aminotransferase; ALT: alanine aminotransferase; ACR: urinary albumin-creatinine ratio
