# Supplementary material for: Reduced gut microbiota diversity in patients with congenital generalized lipodystrophy
Source: Diabetol Metab Syndr. 2022 Sep 24;14:136. doi: 10.1186/s13098-022-00908-8 (PMC9508722; doi:10.1186/s13098-022-00908-8)
Supplement: Supplementary file 5 — Additional file 5. Diversity parameters analyzed by the age, gender, the presence of diabetes, CGL subtype, and the use of metformin or insulin. [file 13098_2022_908_MOESM5_ESM.docx]

**Additional file 5.** Diversity parameters analyzed by the age, gender, the presence of diabetes, CGL subtype, and the use of metformin or insulin

|  | **Age^1^** | **Gender^2^** | **Diabetes^2^** | **CGL subtype^2^** | **Insulin^2^** | **Metformin^2^** |
| --- | --- | --- | --- | --- | --- | --- |
|  | Pre-puberty, puberty, adult | Male or  female | Diabetes: yes or no | Subtype 1 or  subtype 2 CGL | Insulin:  yes or no | Metformin:  yes or no |
| Dominance | 0.413 | 0.191 | 0.208 | 0.336 | 0.255 | 0.281 |
| Richness | 0.386 | 0.460 | 0.805 | 0.459 | 0.532 | 0.189 |
| Shannon | 0.240 | 0.136 | 0.999 | 0.289 | 0.289 | 0.456 |
| Simpson | 0.346 | 0.171 | 0.208 | 0.336 | 0.336 | 0.325 |

CGL: congenital generalized lipodystrophy

Results express in p-value.

^1^ Kruskal-Wallis; ^2^ Mann-Whitney
